# Influence of Zeolite-A Doping and Solvent Mixing Ratio for Electrospun PVDF-Based Membranes

**DOI:** 10.3390/molecules30224353

**Published:** 2025-11-10

**Authors:** Ionut Procop, Viorica Mușat, Elena Maria Anghel, Nicolae Țigău, Felicia Stan, Irina Atkinson, Daniela Cristina Culiță, Alina Cantaragiu Ceoromila, Emanuela Elena Herbei, Radu-Robert Piticescu, Gabriela Ioniță, Alexandru Petrică

**Affiliations:** 1 Department of Materials and Environmental Engineering, Centre of Nanostructures and Functional Materials, Faculty of Engineering, “Dunărea de Jos” University, 111 Domnească Str., 800201 Galati, Romania; ionut.procop.87@gmail.com (I.P.);; 2 Institute of Physical Chemistry-Ilie Murgulescu of the Romanian Academy, 202 Spl. Independenței, 060021 Bucharest, Romania; 3 Department of Chemistry, Physics and Environment, Faculty of Sciences and Environment, “Dunărea de Jos” University, 111 Domnească Str., 800201 Galaţi, Romania; 4 Center of Excellence Polymer Processing, Faculty of Engineering, “Dunărea de Jos” University, 111 Domnească Str., 800201 Galaţi, Romania; 5 Cross-Border Faculty, “Dunărea de Jos” University, 111 Domnească Str., 800201 Galaţi, Romania; 6 National R&D Institute for Non-Ferrous and Rare Metals, 178-184 Biruinței, Pantelimon, 077145 Ilfov, Romania

**Keywords:** fibers, mechanical properties, zeolite-A, electrospun membranes, adsorption

## Abstract

The current study evaluates the characteristics of electrospun PVDF-based membranes doped with zeolite-A in terms of their structural, morphological, thermal, mechanical, hydrophobic, optoelectrical, and adsorption properties. The effects of the DMF–acetone ratio on solvent and zeolite-doping concentration have been evaluated using SEM-EDX, BET, Raman, XRD, DSC-TGA, UV-VIS spectroscopy, contact angle measurements, and mechanical testing. The membranes prepared with solvents low in acetone and increased zeolite content exhibited a higher crystallinity degree exceeding 50%. Zeolite-enriched membranes have a slightly higher content in the α crystalline phase of PVDF when compared to zeolite-free membranes. Electrospinning processing decreased the sample’s subcooling, improving its thermal stability. Zeolite-doping reduced the band gap energy to 1.3 eV from a maximum of 2.7 eV in PVDF membranes. Membranes doped with 3 or 4 wt.% zeolite exhibit improved load-elongation values at break, reaching up to 4.2 N and 47 mm, respectively, and increased flexibility due to their porous structures and the ratio of crystalline to amorphous phases. The membranes adsorbed an MB equilibrium quantity up to 18.5 mg/g and obeyed the pseudo-second-order (PSO) kinetic model within the first 24 h. Thus, the synergistic effect of zeolite content and solvent ratio can effectively adjust the sample’s structure, texture, and properties.

## 1. Introduction

In recent years, synthetic polymer-based membranes have gained attention due to their properties and potential applications [1,2,3,4]. Polyvinylidene fluoride (PVDF) is a versatile polymer used in various applications due to its properties, such as chemical inertness to harsh environments, good thermal stability due to its high melting temperature (lower than 200 °C), glass transition ranging from −29 to −57 °C, hydrophobicity, biocompatibility, and facile processability [1,2,3,4,5]. Its semi-crystalline structure, robust carbon-fluorine (C-F) bonds, and its various crystalline phases (α, β, and γ) provide exceptional properties (chemical inertness to halogens and acidic media, flexibility, and biocompatibility, etc.) and numerous applications. PVDF nanofibers have been widely utilized in environmental remediation applications, including adsorption [6,7], catalytic reactions, and air filtration [3]. Their high surface area-to-volume ratio, especially when engineered with a porous structure, makes them highly effective for capturing contaminants and fine particles, enhancing their performance in environmental remediation applications [8].

Electroactive PVDFs, primarily in their beta- and gamma-phase configurations, exhibit piezoelectric and ferroelectric properties, making them valuable for energy harvesting by converting mechanical energy into electricity. Additionally, they play a significant role in supercapacitor applications, enhancing energy storage capabilities [9,10].

Piezoelectric and sensor applications of materials containing γ-phase PVDF are based on a high piezoelectrical coefficient, accuracy, and sensitivity in detecting thermal, mechanical, and relative humidity changes [11,12]. In the biomedical field, all PVDFs’ polymorphs have been used for drug delivery [1,13] and tissue engineering [14] due to its biocompatibility and controlled release. However, the α-phase of PVDF enhances cell interactions more effectively than the β-phase [15]. PVDF, utilized as a fabric, combines conductivity and flexibility, making it ideal for creating protective clothing and smart clothes embedded with biosensors or flexible electronic components. PVDF is also applied in various fields such as desalination [16], CO_2_ capture [17], and photocatalytic degradation of organic pollutants [18,19,20]. Stretching, compression, electrical poling, and the incorporation of inorganic fillers are among the well-established methods for converting the non-polar PVDF α-phase into the polar PVDF β-phase, enhancing its electroactive properties for various applications [21,22]. Given the weaker piezoelectric behavior of γ-PVDF than β-PVDF, solution-based methods such as contact poling have been recently attempted to convert the γ-phase to the β-phase [23].

Additionally, several techniques have been applied to alter the hydrophobic nature of the favorable α-PVDF phase for water remediation [24]. Nanoparticles with low density and high surface area, such as zeolites [25,26,27,28], serve as excellent candidates for enhancing hybrid membranes. Furthermore, zeolite-based materials have been effectively utilized for dye removal from water [27,28,29]. The zeolite-A, Na_12_[Al_12_Si_12_O_48_]·21H_2_O, containing mostly small eight-membered TO_4_ rings (T is Si and/or Al atoms) in its porous network, can selectively adsorb molecules as CO_2,_ while N_2_ sorption can be prevented by water pore blockage [30]. Although zeolite-enhanced PVDF membranes have promising applications (microplastic and gray water filtration, gas and oil–water separation [27,28]), improving their mechanical properties, hydrophilicity, and fouling resistance remains challenging.

This study aimed to evaluate electrospun zeolite-doped PVDF membranes and how zeolite doping (1–4 wt.%) and different DMF–acetone solvent ratios can tune their crystallinity, morphology, and texture. The influence of these key characteristics has been investigated to optimize band gap energy in semicrystalline structures, as well as to enhance tensile strength. The ongoing research focuses on improving the porous structure, crystalline phases, and thermal, mechanical, and optoelectronic behaviors in order to develop advanced membranes for environmental applications.

## 2. Results and Discussions

### 2.1. Morphology and Composition

**Figure 1:**
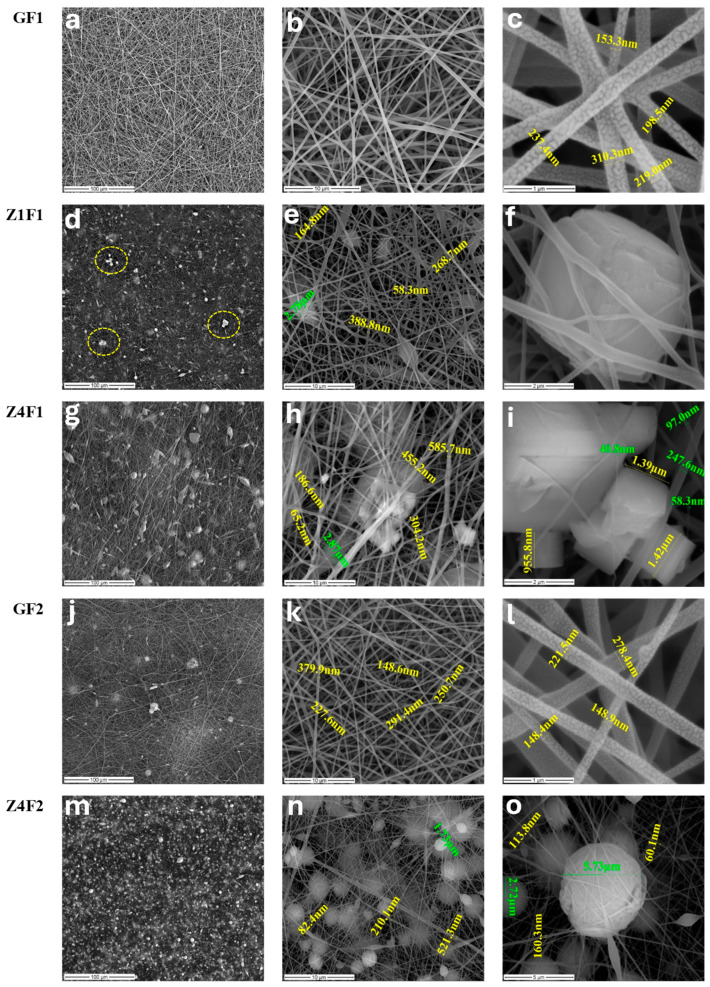
SEM images of the undoped, GF1 (**a**–**c**) and GF2 (**j**–**l**), and zeolite-doped: Z1F1 (**d**–**f**), Z4F1 (**g**–**i**), Z4F2 (**m**–**o**) samples, and PVDF membranes prepared with 70:30 (F1) and 30:70 wt.% (F2) DMF–acetone.

The morphology of the prepared membranes is presented in Figure 1. The SEM micrographs of the GF1 sample prepared with an F1 mixture of solvents (Table 1) show an overall smooth network of fibers.

The fiber’s diameter ranges between 150 and 310 nm with a mean value of about 220 nm (Figure 1c, GF1). At a larger scale, the morphology of the GF2 sample prepared with F2 solvent shows a more compact structure with noticeable beadings (Figure 1j, GF2). Smaller-scale micrographs highlight uniform structures with smooth, well-formed fibers with diameters ranging from 148 to 380 nm (Figure 1k,l, GF2). Research performed by Gee et al. using PVDF 12 wt.% (MW of 450,000–550,000 g/mol) found that a higher DMF concentration (60:40) results in a sample with smoother fibers and fewer beads [31], confirming current results for zeolite-free membranes, GF1 and GF2.

Incorporating 1% zeolite results in a heterogeneous surface, driven by the formation of polymer beads with diameters reaching up to 2.78 μm (Figure 1e, Z1F1) and dispersed or agglomerated zeolite microcrystals (framed details in Figure 1d, Z1F1), randomly distributed among nanofibers (Figure 1e,f, Z1F1). This sample consists of a finer fiber system, with a diameter range of 58–388 nm (Figure 1e, Z1F1). The morphology observed in 4% zeolite–PVDF membranes is even more heterogeneous than for lower zeolite loadings, on the one hand, due to the presence of cubic zeolite microcrystals (Figure 1h,i, Z4F1), but also of polymer beads as bright spheres formed during the electrospinning process (Figure 1n,o, Z4F2). Zeolite aggregation through the fiber network was observed with increased particle content. A broader fiber distribution of PVDF was reported by Lopes et al. [32] due to zeolite agglomeration caused by higher jet instability. The fiber diameter ranges from 65 to 300 nm and 60–200 nm for membranes prepared with the F1 mixture (Figure 1h,i, Z4F1) and the F2 mixture (Figure 1n,o, Z4F2), respectively. The diameters of 455 and 585 nm (Figure 1h, Z4F1) and 521 nm (Figure 1n, Z4F2) correspond to fiber overlaps. The solvent ratio and the zeolite load influence the fiber diameters, beds, and compactness of the obtained membranes, as illustrated in Figure 1.

**Figure 2:**
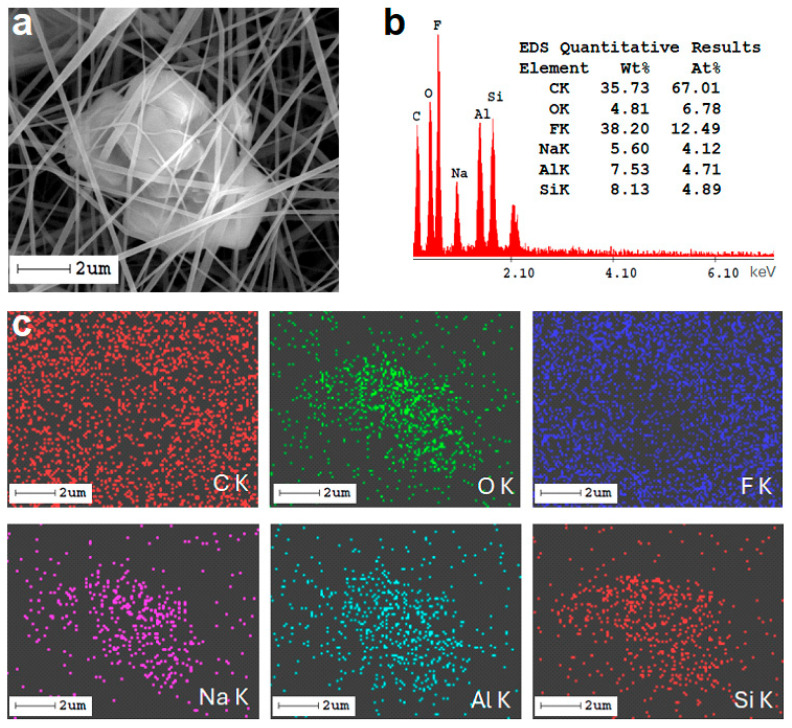
SEM image of selected Z4F1 membrane area (**a**), corresponding EDX spectra with quantitative elemental composition (**b**), and mapping (**c**).

The elemental composition of the zeolite-A-doped PVDF membrane was investigated by energy-dispersive X-ray spectroscopy coupled with SEM (SEM-EDX). Figure 2 shows the results for a selected area (Figure 2a) of the Z4F1-doped membrane. The EDX spectra revealed PVDF (C, O, and F) and zeolite-A (Si, Al, and Na) elements, respectively (Figure 2b). The distribution of the elements Al, Si, O, and Na (Figure 2c) is correlated with the presence of zeolite crystals in the SEM image (Figure 2a).

### 2.2. Structural Properties

#### 2.2.1. Raman Analysis

**Figure 3:**
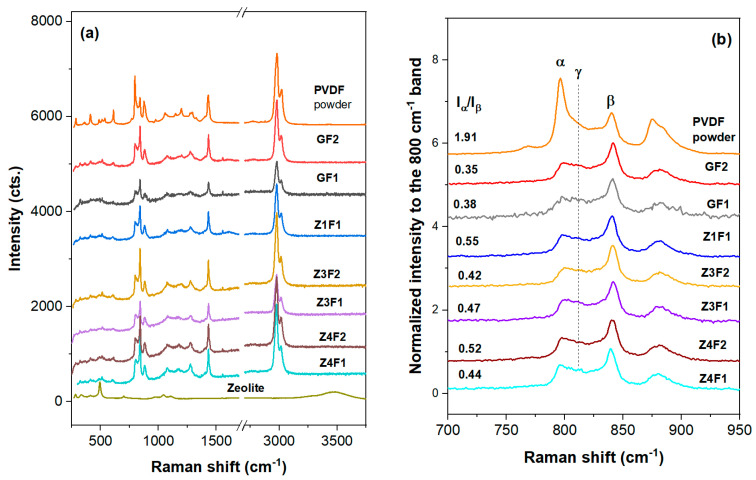
UV-Raman spectra of the zeolite-free GF(1/2), Z(3/4)F2, and Z(1/3/4)F(1/2) membranes within 250–3700 cm^−1^ (**a**) and 700–950 cm^−1^ range (**b**). Spectra of the PVDF and zeolite-A powders were collected for comparison.

The semicrystalline structure and the type of crystalline phases highly influence the properties of PVDF-based membranes. Thus, Raman structural analyses were performed. UV-Raman spectra, illustrated in Figure 3, are dominated by the PVDF spectral features [33], while the zeolite contribution [34] is barely detectable for the rich zeolite-containing samples. Thus, a small band at about 490 cm^−1^ for the rich zeolite samples, Z3F2 and Z4F2, might originate either from zeolite (defect band of the four-membered SiO_4_ rings) [34] or skeletal vibrations of the PVDF identified between 445 and 490 cm^−1^ [35]. Unlike the PVDF powder spectrum, where the α conformers (band at ~800 cm^−1^ for trans-gauche sequences, tg) prevail, the membrane spectra with or without zeolite show an enhanced amount of the β conformers (main band at 840 cm^−1^). The intense band at about 840 cm^−1^ belongs to PDVF in forms I (all-trans and all-t of the β-phase) and III (three trans and one gauche, t3g, in the γ-phase), but is strong in form I [33,35]. The weak 811 cm^−1^ band enables the t3q sequence identification in the γ-phase. Otherwise, membrane spectra in Figure 3 are rich in trans-zig-zag sequences (form I) of β-phase with some t3g and tg content, e.g., γ- and α-phases. To identify a specific phase, all phase features must be present in the spectra. Thus, except the 840 cm^−1^ band, some identification bands for the β-phase are present in the 900–1300 cm^−1^ region, e.g., 1280 cm^−1^, v (CF_2_), and 1434 cm^−1^, δ (CH_2_). Smaller crystallite and/or increased amorphous content of the membranes is depicted by a bigger FWHM of the Raman bands (see Table S1). The Z1F1 sample exhibited broader bands at 800 and 840 cm^−1^ (Figure S1 and Table S1), corresponding to its thinner fiber texture and the presence of large cubic polymer beads.

The intensity of the 799/840 or α/β ratio [36] shows the tg/all-t sequence distributions in the studied membranes (Figure 3b and Table S1). A subunit I_α_/I_β_ ratio indicates a major β-phase for all the membranes discussed in this work. Moreover, increasing zeolite content to 1 wt.% (ZF1) trigged the increase in the α/β ratio to 0.55, while this ratio decreases for Z3F1 (0.47) and Z4F1 (0.43). The observed non-monotonic changes with increasing zeolite content are likely due to the noticeable 811 cm^−1^ peak, associated with the γ-phase, in samples richer in zeolite, e.g., Z(3/4)F1. The I_α_/I_β_ ratio increases from GF2 (~0.35) to Z3F2 (~0.42) and Z4F2 (~0.52). A slightly bigger ratio was recorded for GF1 than for GF2, meaning that the solvent used in membrane preparation might have a small influence on the PVDF conformers and tailoring membrane properties. Since a rich β-phase is present in the investigated membranes, piezoelectric behavior is expected for the membranes in this work.

#### 2.2.2. XRD Analysis

**Figure 4:**
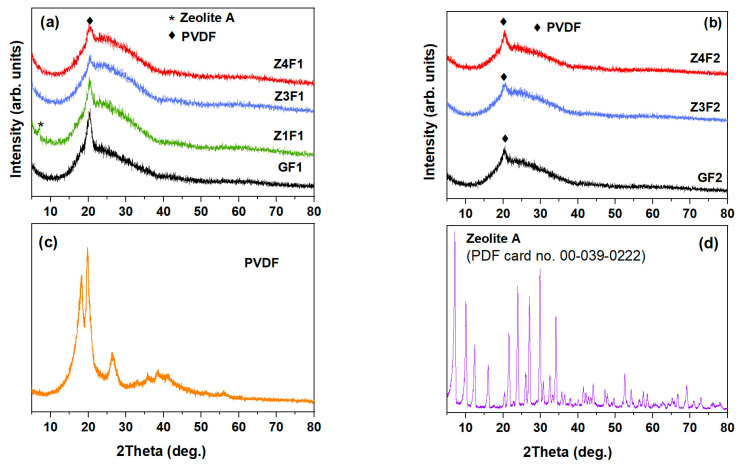
XRD of zeolite-doped PVDF membranes (**a**,**b**), PVDF powder (**c**), and zeolite nanoparticles (**d**).

Figure 4 shows the X-ray patterns of the investigated membranes, PVDF powder, and zeolite-A as reference samples. The most intense crystalline peaks within 17–27° correspond to the α-, β-, and γ-phases of PVDF (Figure 4c), respectively [37,38,39]. The PVDF polymer chains are organized in different structures (conformers). Thus, α-phase with diffraction peaks at 17.66°, 18.30°, 19.90°, and 26.56° is non-polar due to the antiparallel arrangement of its chains and does not encounter any positive or negative charge. The material in this phase is used for its mechanical flexibility [39]. The diffraction peak at 20.26° belongs to the β-phase polar polymer chains arranged into a trans-zig-zag structure. γ-phase shows a diffraction peak around 20.04° due to a mix of molecular arrangements intermediate between those of α-phase and β-phase with some polar properties and some flexibility, i.e., three trans and one gauche sequence (t3g) [10,38] In Figure 4b, the bottom graph represents zeolite-A nanoparticle powder and it serves as a reference, showing peaks at around 10° and 21–34°, which are typical for this structure [40,41].

Figure 4a,b show the X-ray diffraction patterns of the investigated samples, respectively. The zeolite-containing and zeolite-free membranes show a less intense peak profile when compared to PVDF powder. This effect could be attributed to the presence of zeolite and the electrospinning process, which influence both the proportion of the β-phase and the degree of crystallinity [11,27]. A less crystalline GF2 sample than the GF1 sample is noticed in Figure 4a,b. The XRD analyses indicate a partial loss of crystallinity for all, G(F1/F2), and zeolite-doped PVDF membranes, compared to the PVDF powder (see Figure 4a).

Deconvolution of the XRD patterns, focusing on the main crystalline phases (α-phase, β-phase, and γ-phase), was performed for PVDF powder, pristine PVDF (GF1), and Ze-doped PVDF (Z4F1) fibrous samples, as illustrated in Figure S2. Both membranes were obtained with the F1 solvent. According to the most intense halo between 10° and 40° of the Z4F1 diffractogram in Figure S1, its enhanced amorphous phase fraction was caused by the richer zeolite content. Moreover, a diminished β-phase content in the Z4F1 was noticed. Since the electrospinning process has been reported to trigger an increase in the crystallinity degree and β-phase content [11], peculiar behavior of the Z4F1 could be attributed to the zeolite content and solvent (F1) used during the fibrous samples’ preparation [42,43].

### 2.3. Textural and Surface Properties

The nitrogen adsorption–desorption isotherms of the PVDF and PVDF/zeolite-A membranes (Figure S3) were recorded to determine their textural characteristics. The isotherms show very low adsorbed nitrogen volumes, indicating that the membranes are weakly porous. The textural parameters summarized in Table 1 confirm this observation. All samples exhibit low specific surface areas (<11 m^2^g^−1^) and total pore volumes (<0.063 m^2^g^−1^). Nevertheless, minor differences in the calculated parameters may reflect changes in the membranes’ morphology induced by solvent composition and zeolite loading [42,43].

Shimada et al. [44] reported that the surface wettability of semicrystalline PVDF is highly controlled by competing molecular orientation and surface properties. Typically, surface wettability is estimated by contact angle measurements. The perpendicular orientation of the c-axis in β-phase conformers could influence the wettability characteristics of PVDF membranes, potentially affecting their hydrophobicity or hydrophilicity depending on the structural alignment and intermolecular interactions [44]. A contact angle of 131.3° (Figure S4) of the control GF1 fibrous sample indicates its hydrophobicity. Thus, although the undoped membranes of both series have close values (around 130–132°, Figure S4), while the values increase slightly to 137–138° for the membranes of the F1 series (Figure S4a), it decreases to 103 (Z3F2) and rises slightly above 110 (Z4F2) degrees for those of the F2 series (Figure S4b). Although the zeolite-doped materials in set F1 exhibit similar contact angle values, the I_α_/I_β_ ratio in the Raman spectra (0.55, 0.47, and 0.44 in Figure 3b) decreases with increasing zeolite content (1, 3, and 4 wt.%), suggesting that the solvent used during preparation might have a more significant influence. Conversely, for the F2 sample set, where the I_α_/I_β_ ratio increases with increasing zeolite amount, both the solvent used and the amount and packing mode of the zeolite (Z4F2 micrographs in Figure 1) seem to influence the contact angle values. The different behavior was due to the solvent used for preparation, zeolite content, and zeolite wrapping by PVDF fibers [32].

### 2.4. Thermal Analysis

**Figure 5:**
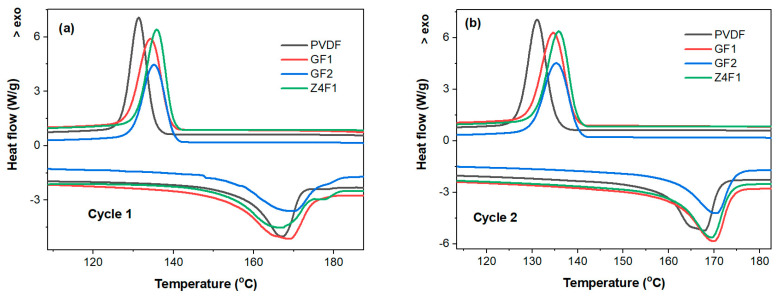
mDSC heating–cooling first (**a**) and second (**b**) cycles of selected electrospun membranes.

The modulated thermal analyses (mDSC and mTGA) were performed for membrane selection (Figure 5 and Figure S5, respectively). The DSC heating–cooling curves for two successive cycles are presented in Figure 5a,b. The melting temperature (T_m_) of pure PVDF powder at 166.5 °C is shifted to slightly higher temperatures of 168.7 and 168.9 °C upon the electrospinning deposition of the undoped membranes GF1 and GF2, respectively, and decreases by ~1 °C when adding 4 wt.% zeolite for Z4F1 (Figure 5a). A bimodal melting peak was observed, especially for samples GF1 and Z4F1. The origin of the multiple melting peaks in PVDF-based materials remains a subject of debate [45,46]. Three explanations are available for this behavior: PVDF polymorphism (α-, β-, and γ-phases), the melting–recrystallization phenomenon during sample heating, and morphological modifications in lamellar thickness and crystal imperfections [45]. Based on a polymorphous mechanism, the first melting temperature of 167 °C corresponds to the β-phase, while the one at 171 °C belongs to the α-phase, and the melting peak at 180 °C originates from the γ-phase [1,46]. Further DSC investigations at various heating–cooling rates are required to establish the melting mechanisms of the PVDF membranes under discussion. These T_m_ values of the electrospun membranes are approximately 10 °C higher compared to those reported by Shen et al. for membranes doped with comparable zeolite-A concentrations (4–10%) obtained by casting [47]. The cooling curves show only a single exothermal peak corresponding to β-phase recrystallization, with maximum crystallization temperatures (T_c_) of 134.2 °C and 135.06 °C for the undoped membranes (GF1 and GF2, respectively), and 135.6 °C for the doped Z4F1 (Table S3). The subcooling (T_m_-T_c_) decreased compared to the powdered polymer. Although both temperatures increased, the increase in T_m_ values was twice that of the T_c_ ones, indicating an increase in thermal stability. As expected, after erasing the thermal history in the first heating–cooling cycle, changes in the peak temperature of 1–1.5 °C and the curve shapes were also observed between the melting and crystallization temperatures for the second heating–cooling cycle (Figure 5b).

Although the solvent appears to have a minor impact on the PVDF conformers in the electrospun PVDF-based membranes under discussion, the crystallinity degree, determined from the integrated melting effect of the electrospun membranes, exhibits a more pronounced variation [48] (Table S2). Thus, Z4F1 (54.76%) and GF1 (53.65%) encountered a higher crystallinity degree than GF2 (43.12%) and PVDF powder (47.57%). Moreover, the GF2 fibrous sample obtained with an acetone-rich solvent is less crystalline than PVDF powder. Recently, it has been reported that polymorph types, as well as oriented amorphous fractions that exist at the crystalline–amorphous interface, can influence the piezoelectric response of PVDF materials [49].

The thermogravimetric (mTGA and mDTGA) curves (Figure S5) reveal two weight-loss steps of membranes’ decomposition, with maximum reaction rates at 431.4 and 432.9 °C (first step) and at 492.92 and 481.82 °C (second step) for GF1 and Z4F1, respectively. This highlights that the zeolite-A addition slightly improved the thermal stability in the first stage but decreased it in the second stage. Slightly smaller weight loss for Z4F1 (97.71%) than for GF1 (99.91%) was due to zeolite residue. Overall, the undoped and 4 wt.% zeolite-doped membranes show quite similar behavior in terms of melting and recrystallization temperatures. However, zeolite doping causes a 10 °C decrease in thermal stability above 450 °C. This originated from the varied phase structure of the polymer fibers.

### 2.5. Optical Properties

**Figure 6:**
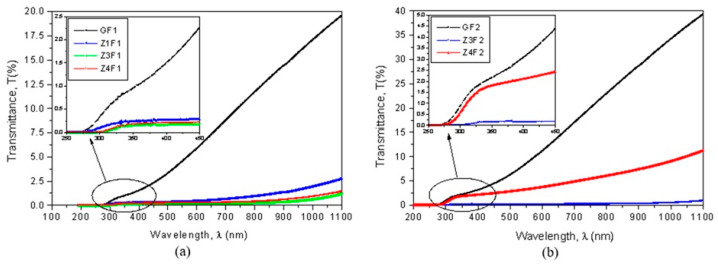

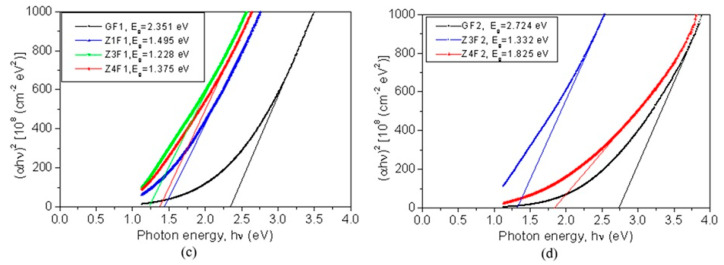
Transmittance as a function of wavelength (**a**,**b**) and Tauc’s plots (**c**,**d**) for evaluation of optical band gap energy (E_g_).

The optical properties of the two series of membranes prepared with F1 and F2 solvents indicate small optical transmittance in the UV-VIS-NIR domains (Figure 6a,b). The GF1 and GF2 membranes reached the highest values and the thinner-wired sample obtained from the more volatile F2 mixture solution (Figure 6b) had significantly higher transparency than that prepared with F1 (Figure 6a). Optical transmission decreases as zeolite content increases.

The capability of these membranes to be used for developing photocatalysts by generating electron (e^−^) and hole (h^+^) pairs can be evaluated based on their optical bandgap energies. Their bandgap energies (E_g_), calculated using Tauc’s plots, are shown in Figure 6c,d. The undoped GF1 sample has a bandgap energy of 2.351 eV. With the addition of zeolite, the E_g_ value decreases to 1.495 eV (Z1F1) and reaches 1.375 eV for the Z4F1 sample. The GF2 sample has a bandgap energy of 2.724 eV, which is higher than that of GF1. By doping with zeolite, the bandgap decreases up to 1.332 eV for Z3F2 and increases to 1.825 eV for Z4F2. According to these bandgap energy values [50], these composites are well-suited as photocatalysts in the degradation of organic pollutants.

### 2.6. Mechanical Properties

**Figure 7:**
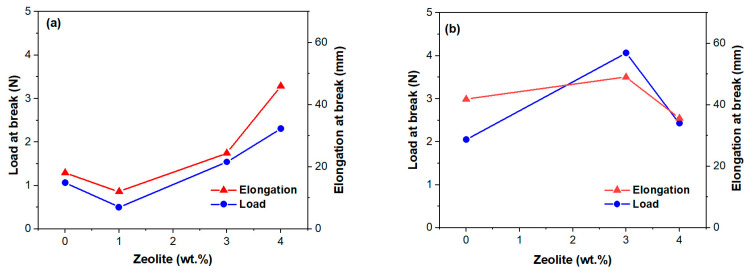
Tensile properties of the investigated membranes of the F1 (**a**) and F2 (**b**) series versus zeolite content.

Efforts to improve the performance of PVDF membranes have generally focused on their piezoelectric properties and less on their mechanical properties [51], although these are essential for their durability. The data in Figure 7 shows the mechanical performance of the membranes with and without the addition of zeolite. Preparation methods, fiber thickness, and zeolite load are a few factors influencing the mechanical behavior of the zeolite-doped PVDF membranes. Thus, zeolite-doped membranes exhibit improved mechanical performance (Figure 7) mainly due to their thicker fibers [51]. The GF1 sample shows moderate load at break and elongation compared to the zeolite-doped membranes (Figure 7a). It should be noted that the load at break corresponds to the maximum load exhibited by the membranes. Sample Z1F1 exhibits a notable decline in mechanical properties compared to GF1. This behavior was driven by the Z1F1 fiber’s finer structure (Figure 1), setting it apart from the other membranes in the F1 set. In contrast, the Z3F1 sample exhibited enhanced mechanical properties compared to Z1F1 and GF1. Furthermore, the Z4F1 sample has the highest load and elongation at break values, indicating that increased zeolite content enhances resistance to mechanical deformation, reinforcing its structural integrity. Hence, zeolite content and fiber diameter govern the sequence of the load and elongation at break values, e.g., Z4F1 > Z3F1 > GF1 > Z1F1. This sequence is opposite to the one of the I_α_/I_β_ ratio (Figure 3b) for the zeolite-doped F2 sample set, meaning the richer β-phase content might favor improving the mechanical parameters.

Another variable factor is the solvent. The GF2 and Z3F2 membranes, with denser morphologies (Figure 1) and lower crystallinity degrees, exhibited higher load and elongation break values compared to GF1 and Z3F1, respectively. However, the fibrous sample doped by 4 wt.% Ze (Z4F2) behaves differently. Thus, both parameters decrease, interestingly reaching very close values to each other (Figure 7b). It is worth noting that while the load at break value for Z4F2 approaches that of the Z4F1 sample, the elongation break is two times lower than that of Z4F1 (Figure 7a). Based on these results, zeolite contributes to better mechanical properties of the doped membranes.

Analogous to He et al. [11], the 3–4% addition of zeolite particles improved the membranes’ mechanical properties. Chan et al. [52] also confirmed improvements in tensile strength at low zeolite doping (0.5%) but a diminishing elongation due to zeolite overloading and reduced intermolecular interactions of the polymer chains. At the same zeolite content, higher elongation of Z4F1 than Z4F2 might be related to its richer β-phase content (Raman intensity ratio, I_α_/I_β_, of 0.44 and 0.52, respectively). The dissimilar β-phase content was very likely caused by the different solvents used in preparation. Moreover, lower tensile parameters in Figure 7b for the 4 wt.% of zeolite point out filler overloading in the denser Z4F2 membrane.

### 2.7. Methylene Blue Adsorption by Electrospun Membranes

A rapid increase in the first 2 h of MB sorption on the membranes is depicted in Figure S6, showing MB adsorption efficiency as a function of time. This is due to the active site available for MB adsorption. No adsorption efficiency exceeds 88% (Z4F1 in Figure S6a). The greater the proportion of the β-phase (Figure 3b), the higher the adsorption capacity (Figure S6) of the zeolite-containing samples. This is due to the stronger interaction of the dye molecules with the polar phase, i.e., the beta phase of PVDF [53]. Porosity and pore size of the PVDF/MWCNT materials influence the equilibrium adsorption quantity (2.4, 4.4, and 5.68 mg/g for untreated and ultrasound-treated membranes [54]). A higher adsorption capacity was reported for zeolite materials, e.g., 88.51 mg/g (NaZ), 68.04 mg/g (HZ), and 41.37 mg/g (DE) using a 110 mg/L MB solution [55].

**Figure 8:**
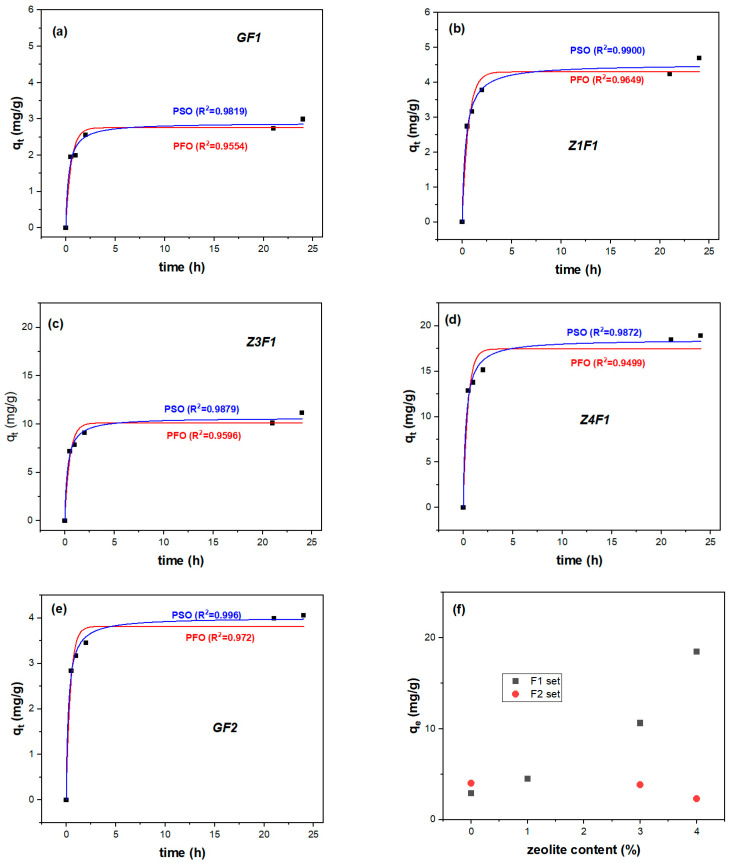
PFO and PSO adsorption kinetic fitting models of GF1 (**a**), Z1F1 (**b**), Z3F1 (**c**), Z4F1 (**d**), GF2 (**e**), and equilibrium adsorption quantity of all membranes (**f**).

To analyze MB adsorption on electrospun membranes within 0–24 h, two kinetic models (pseudo-first-order, PFO; pseudo-second-order, PSO) were employed (Figure 8 and Table 2). Thus, MB absorption follows the PSO model within the first 24 h in accordance with its correlation coefficients. Increasing the zeolite content in the F1 membranes resulted in enhanced adsorption compared to GF1 and the other PVDF membranes [53]. Unlike the F1 set materials, the adsorbed MB on the Z3F2 and Z4F2 samples declines compared to zeolite-free GF2 (Figure 8f). Hence, the better adsorption performance of Z3F1 and Z4F1 samples is evident compared with the F2 counterparts.

These results indicate that the pseudo-second-order (PSO) model better describes the adsorption kinetics, suggesting that chemisorption is the dominant rate-controlling mechanism. According to this model, the adsorption rate depends on the interaction between the PVDF fiber and/or zeolite adsorbents and the MB dye by covalent bonding and/or ion exchange [56].

The kinetic study for initial BM concentration in solutions ranging from 20 to 80 mL/L highlighted differences in the adsorption mechanism on the Z4F1 membrane (Figure S7) and in the percentage of MB extracted from the solution. It was also observed that equilibrium adsorption was attained faster at lower MB concentrations. Thus, at 20 mg/L, the pseudo-first-order (PFO) model better described MB dye adsorption, with removal percentages reaching 97% for 20 h. The PFO model indicates a physisorption process, associated with weaker adsorption interactions, as van der Waals forces. The shorter time stabilization of the adsorption equilibrium at low MB initial concentration is a consequence of a relatively higher concentration of active adsorption sites of the PVDF material with respect to the limited number of available MB molecules, resulting in a rapid adsorption [57].

At higher concentrations of 40, 60, and 80 mg/L, closer to one, correlation coefficients for the non-linear kinetics with the pseudo-second-order (PSO) model indicate a chemosorption process, dependent on both MB concentration and the number of available active sites on the adsorbent. Patel et al. demonstrated electrostatic interactions between the PVDF polar molecules and the positively charged quaternary ammonium groups in MB dye by DFT simulations, validated with experimental data/PSO model [58]. At 40–80 mg/L MB concentrations, the adsorption efficiency decreased to 61, 54, and 55%, respectively (Figure S7). Similar decreases have been observed in other studies, attributed to a higher ratio of MB molecules to available adsorption sites. This shift renders the adsorption process more intricate, thereby prolonging the time to reach equilibrium. Before saturation of the adsorbent, methylene blue (MB) adsorption occurs through multiple stages, including diffusion to the sorbent’s outer surface, interaction on the surface itself, and diffusion into pores [57].

## 3. Materials and Methods

Acetone (CAS No. 67-64-1, 99.92%) from Merck KGaA, Darmstadt, Germany, dimethylformamide (DMF), CAS No. 68-12-2, 99.8% from Honeywell, Seelze, Germany, PVDF of Mw = 140,000 g mol^−1^ (donated by Foshan Qingzi Precision measurement and control technology Co., Ltd., Foshan, China), and zeolite 96096 (CAS No. 1318-02-01 Sigma-Aldrich, Darmstadt, Germany) were used for sample preparation.

To obtain an electrospun PVDF 12 wt.% sample, a solution (10 g) was formulated by mixing 1.2 g of PVDF powder with 8.8 g of solvent. The polymer solution was prepared using two mixtures with DMF and acetone as solvents. The first mixture, designated as F1, contained 70 wt.% DMF and 30 wt.% acetone, while the second mixture, F2, comprised a 30:70 wt.% ratio of DMF to acetone (Table 1). Therefore, two series of samples were prepared, using the mixtures F1 and F2. Within these series, the zeolite concentrations varied between 0.5 and 4 wt.%. The PVDF powder (1.2 g) was gradually dissolved in an 8.8 g mixture of F1 or F2 solvents, with continuous stirring for three hours to prevent clumping and ensure uniform dispersion. The temperature of the polymer mixture gradually rose to 50 °C and was maintained at that level for an additional 30 min. Once cooled to room temperature, a portion of these solutions was utilized to produce the reference membranes (GF1 and GF2). The two PVDF solutions were doped with zeolite at concentrations ranging from 0.5 to 4 wt.%, a process carried out at room temperature. Based on the zeolite content, the resulting solutions were named as follows: Z1F1 (1 wt.%), Z3F(1/2) (3 wt.%), and Z4F(1/2) (4 wt.%), as listed in Table 1. Each solution was loaded into a 5 mL syringe with a 22-gauge needle, ensuring the absence of air bubbles, and subsequently electrospun using an E03-001 (Foshan, China) device. Table 1 shows sample coding and its textural parameters [6,7].

The sample was deposited on a static flat ground collector covered in non-stick paper (baking paper). The delivery pump was set to 1.00 mL/h and the voltage was set at 12 kV. The distance to the collector was 10 cm, at a temperature of 28 °C, and the room’s humidity was about 35%. The generated electrical field and the flow were slightly adjusted to achieve a stable Taylor Cone. Fibers were deposited for approximately 90–100 min. The resulting sample was removed from the flat collector and dried at room temperature for 24 h, facilitating solvent evaporation under a ventilated laboratory hood.

The characterization of the membranes encompasses the analysis of their morphology, chemical structure, crystalline phases, porosity, surface parameters, optical absorption spectra, bandgap energy, thermal stability, and mechanical behavior.

For SEM-EDX (scanning electron microscopy—energy dispersive X-ray), the SEM microscope (Quanta 200 FEI, Brno, Czech Republic) was coupled with an energy dispersive X-ray (EDX). SEM analysis was performed after the sample was vacuum-coated with a thin layer (4 nm) of gold alloy using an SPI-Module™ sputter coater. The microscope was operated at 15 kV (accelerating potential). SEM images were taken at magnifications ranging from 200× to 100,000×, providing detailed images of the sample’s structure at 500, 10, and 1 μm scales. The EDX system provided elemental composition and distribution for PVDF, zeolite, and additives in the fibrous samples.

Raman spectra of the obtained electrospun membranes, PVDF, and zeolite-A powders were collected using a LABRam HR800 spectrometer (Horiba France SAS, Palaiseau, France). A He-Cd laser (325 nm laser line) excited the samples through an Olympus 40× NUV/0.47 microscope objective (Tokyo, Japan). A CCD detector and 2400 g/mm gratings enabled high-resolution measurements.

The nitrogen adsorption–desorption technique was used to determine the textural properties of the materials. Nitrogen adsorption–desorption isotherms were obtained using a Micromeritics ASAP 2020 analyzer (Norcross, GA, USA) at 77 K. The BET equation was applied to calculate the specific surface area while the total pore volume was estimated from the amount adsorbed at the relative pressure of 0.99.

The X-ray diffraction (XRD) patterns were obtained using a Rigaku Ultima IV diffractometer (Rigaku Corp., Tokyo, Japan) with Cu Kα radiation (λ = 0.15406 nm) within a 2θ range of 5° to 80° at a scanning speed of 2°/min and a step size of 0.02°. Data analysis was performed using Rigaku’s PDXL software (Version 1.8), which was connected to the ICDD database.

Contact angle measurements were performed using a Drop Shape Analyzer DSA100 KRÜSS (Hamburg, Germany) to assess the sample wettability, determining whether it exhibits hydrophobic or hydrophilic properties. This parameter is important for optimizing water purification and filtration applications. The experiments involved a wetting agent (double-distilled water) and controlled environmental parameters (temperature 20 ± 1 °C). Images were captured ~1 s after the wetting agent droplet deposition. The results were analyzed using advanced software, with an average of three determinations conducted for each sample to ensure accuracy and reliability.

Tensile tests were performed to investigate the effect of fillers on the mechanical behavior of the electrospun membranes (e.g., load and elongation at break). Tensile tests were conducted on a universal testing machine M350–5AT (Testonic Co Ltd., Rochdale, UK) at room temperature (23 ± 2 °C). The crosshead displacement was set to 1 mm/min, and the gauge length to 50 mm.

Modulated differential scanning calorimetry (mDSC) coupled with modulated thermogravimetric (mTGA) and derivative thermogravimetric (mDTGA) measured the temperature, mass loss, and reaction rate associated with sample disintegration during heating, providing information about chemical stability at elevated temperatures. The modulated curves (mTGA and mDSC) were recorded using Q20 TA and Q5000IR Thermal Analysis devices (Instruments, New Castle, DE, USA) in synthetic air (5.0 purity) at a heating rate of 10 K/min.

UV–VIS spectra were collected within the 250–1200 nm range with a Perkin Elmer Lambda 950 UV/VIS/NIR spectrophotometer (PerkinElmer Ltd., Beaconsfield, UK). The band gap energy (E_g_) of the membranes was calculated using Tauc’s equation [59] to evaluate their suitability for optoelectronic and photocatalysis applications [60].

To investigate the adsorption properties, fibrous samples were placed in an 8 mL solution of methyl blue (MB) at 25 mg/L and kept in the dark to monitor MB discoloration. The amount of MB adsorbed per unit of adsorbent (q_t_) and the dye adsorption efficiency (η) at the sampling time were calculated using the following equations:

(1) $$\eta \left(\%\right)=\frac{c_{0}-c_{t}}{c_{0}}$$

(2) $$q_{t}=\frac{c_{0}-c_{t}}{m}\times V$$

where *c*_0_ and *c_t_* are the dye concentrations (mg/L) before adsorption and at time t. V is the volume of the dye solution and *m* represents the weight of the absorbent used (g).

## 4. Conclusions

The study highlights the significant impact of the solvents’ ratio and the addition of zeolite content (1.0 to 4.0 wt.%) on the structural and functional properties of the successfully electrospun PVDF-based membranes. The current findings indicate that high-molecular-weight PVDF (450,000–550,000) in a solvent system with a lower evaporation rate, combined with a low polymer concentration (12%), results in the formation of fewer beads. This suggests that solvent evaporation dynamics and polymer concentration play crucial roles in determining the sample’s morphology. Larger diameter fibers ranging between ~60 and 300 nm were obtained for zeolite-doped membranes, especially at a higher zeolite content. Bigger fiber diameters (450–580 nm) for 4 wt.% zeolite doping appear like conglutination/twin fibers.

The combination of the highest zeolite load and the F2 solvent ratio (Z4F2) yields dense, compact membranes with a higher ratio of α/β conformers, enhanced adsorption capacity, and is optimal for adsorption applications. Increasing zeolite content reduces transparency and decreases the bandgap energy below 2 eV, which is suitable for the photocatalytic degradation of organic pollutants. The incorporation of zeolite triggers an increase in load-elongation values at break, reaching 4.2 N and 47 mm, respectively, while also enhancing flexibility and porosity. These improvements contribute to superior filtration efficiency, positioning these composites as strong candidates for advanced separation technologies. Optimizing the distribution of zeolite remains a challenge that can be considered for improving the performance of the membranes.

For the F1 membrane series, increasing the zeolite content resulted in continuously enhanced equilibrium adsorption, from 2.9 (GF1) to 18.5 (Z4F1) mg/g. The pseudo-second-order (PSO) model better describes adsorption kinetics, suggesting interactions through covalent bonding and/or ion exchanges.

## Figures and Tables

**Table 1:** Sample membrane coding and its textural parameters.

Code	DMF–Acetone	Zeolite (wt.%)	S_BET_ (m^2^/g)	Total Pore Volume (cm^3^/g)
GF1	70:30	0	9.5	0.063
Z1F1	70:30	1	7.1	0.024
Z3F1	70:30	3	7.5	0.023
Z4F1	70:30	4	8.5	0.020
GF2	30:70	0	9.7	0.024
Z3F2	30:70	3	10.8	0.025
Z4F2	30:70	4	8.9	0.025

**Table 2:** Kinetic model parameters for the time-dependent MB adsorption on the Ze-PVDF membranes.

Model	Parameters	GF1	Z1F1	Z3F1	Z4F1	GF2
PFO $q_{t}=q_{e}\left[1-e^{-k_{1}t}\right]$	q_e_ (mg/g)	2.75373 ± 0.1560	4.2968 ± 0.2213	10.1003 ± 0.5387	18.4422 ± 1.0284	3.8112 ± 0.1642
	k_1_ (min^−1^)	1.8518 ± 0.4416	1.6066 ± 0.3254	1.9918 ± 0.4646	2.1226 ± 0.5673	2.3428 ± 0.4862
	R^2^	0.9554	0.9649	0.9596	0.9499	0.9720
PSO $q_{t}=\frac{q_{e}^{2}k_{2}t}{1+q_{e}k_{2}t}$	q_e_ (mg/g)	2.8828 ± 0.1142	4.5124 ± 0.1339	10.6300 ± 0.3419	18.4701 ± 0.6086	4.0091 ± 0.0727
	k_2_ (g/mg/min)	1.9339 ± 0.4161	0.6068 ± 0.0100	0.3299 ± 0.0637	0.1950 ± 0.0388	1.0651 ± 0.1365
	R^2^	0.9818	0.9900	0.9879	0.9872	0.9996

## Data Availability

The original contributions presented in this study are included in the article/Supplementary Materials. Further inquiries can be directed to the corresponding authors.

## Supplementary Materials

The following supporting information can be downloaded at: https://www.mdpi.com/article/10.3390/molecules30224353/s1, Figure S1. UV Raman spectra of the GF(1/2), Z(3/4)F1, and Z(3/4)F2 membranes within a 700–950 cm^−1^ range. The spectrum of the PVDF powders (black) is presented as a reference. Figure S2. Deconvoluted XRD patterns of the PVDF polymer powder, zeolite-free PVDF sample (GF1), and zeolite-doped PVDF (Z4F1) membranes. Figure S3. Nitrogen adsorption–desorption isotherms for undoped (GF1 and GF2) and zeolite-doped (Z1F1, Z3F1, Z3F2, Z4F1, and Z4F2) membranes. Figure S4. Contact angle measurements of the F1 (a) and F2 (b) series. Figure S5. Modulated thermogravimetric curves of the zeolite-free (GF1) and zeolite-doped (Z4F1) membranes. Figure S6. MB adsorption efficiency on the (a) F1 membranes and (b) F2f membranes. Figure S7. Adsorption efficiency of the Z4F1 fibrous sample at various MB concentrations (inset stands for 40 mg/L MB adsorption kinetics on the Z4F1 sample). Table S1. Results of fit parameters (peak position/FWHM) and assignments for the membranes within the 700–950 cm^−1^ range. Table S2. DSC-fitted parameters (melting data) and crystallization degree (χ_m_) of the zeolite-free PVDF-based, GF(1/2), and Z4F1 fibrous samples collected during a second heating cycle; Table S3. Melting and crystallization points (DSC data) collected during two successive heating–cooling cycles. Ref. [61] is cited in the Supplementary Materials.
