# Supplementary material for: Influence of Zeolite-A Doping and Solvent Mixing Ratio for Electrospun PVDF-Based Membranes
Source: Molecules. 2025 Nov 10;30(22):4353. doi: 10.3390/molecules30224353 (PMC12654574; doi:10.3390/molecules30224353)
Supplement: Supplementary file 1 [file molecules-30-04353-s001.zip › molecules-3934538-supplementary.pdf]

Supplementary Information

Influence of Zeolite-A doping and solvent mixing ratio for electrospun PVDF membranes

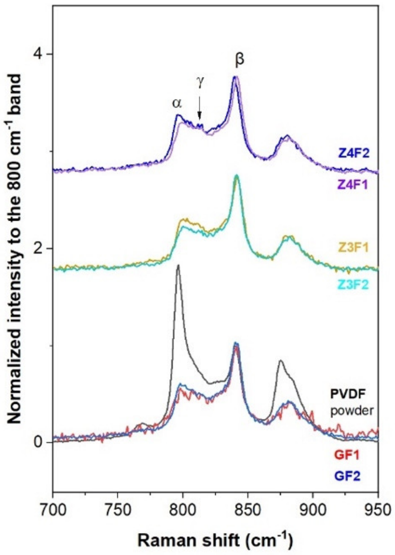

Figure S1. Raman spectra of the GF(1/2), Z(3/4)F1, and Z(3/4)F2) samples within and 700-950 cm^{-1} range. The spectrum of the PVDF powders (black) is presented as a reference.

Mixtures of PVDF polymorphs are noticeable in Figure S1. The β-phase prevails in the Raman spectra of the samples prepared in this work compared with the PVDF powder containing a higher amount of α-phase.

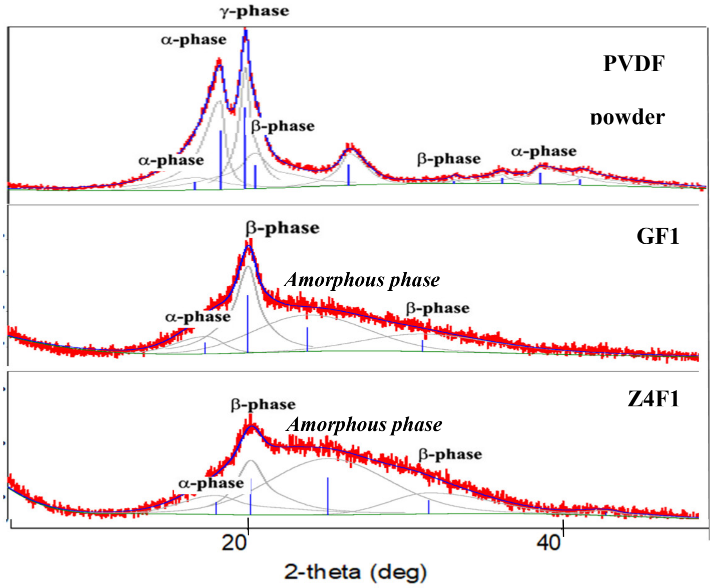

Figure S2. Deconvoluted XRD patterns of the PVDF polymer powder, Zeolite-free PVDF (GF1), and zeolite-doped PVDF (Z4F1) samples. The amorphous phase is also depicted in the obtained samples.

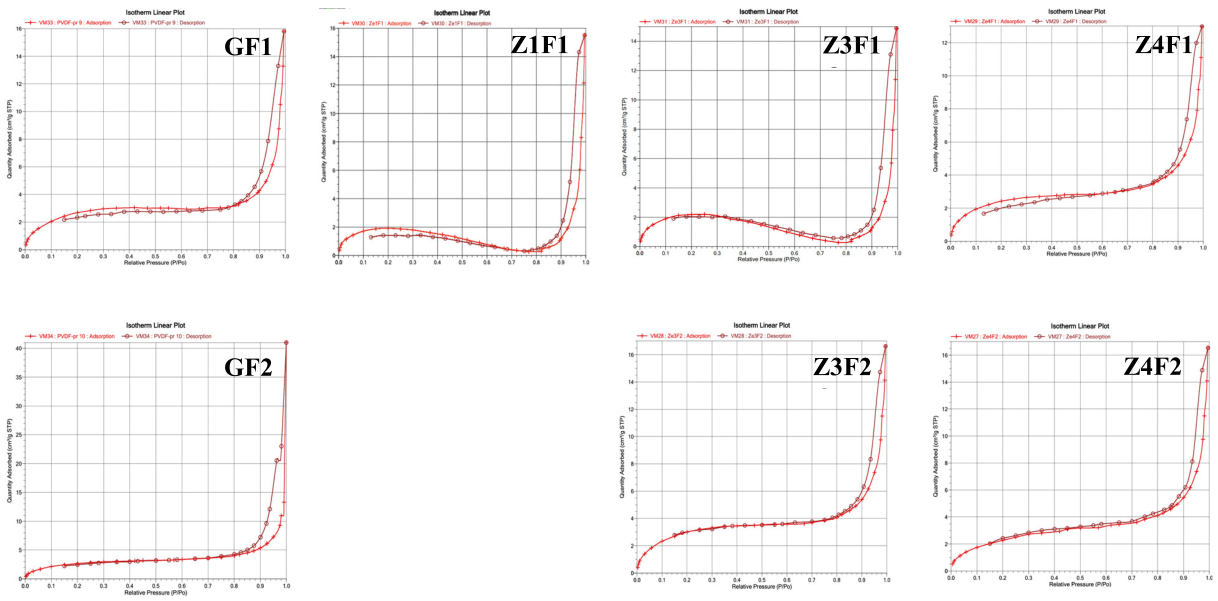

Figure S3. Nitrogen adsorption-desorption isotherms for undoped (GF1, GF2) and zeolite-doped (Z1F1, Z3F1, Z3F2, Z4F1 and Z4F2) samples

(a) (b)

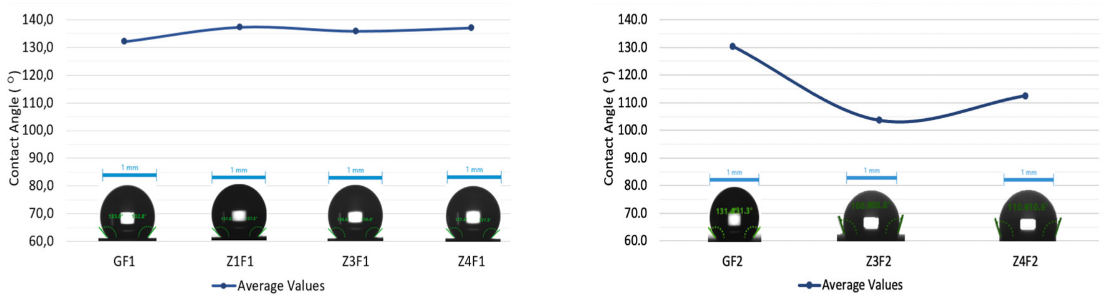

Figure S4. Contact angle measurements of the F1 (a) and F2 (b) series.

The contact angle seems to be insensitive to the beta-PVDF and zeolite content in the F1 set of materials (Figure S4a). Conversely, when the alpha-PVDF phase and amorphous content increased with zeolite load to 3 wt.%, the contact angle decreased slightly (Figure S4b).

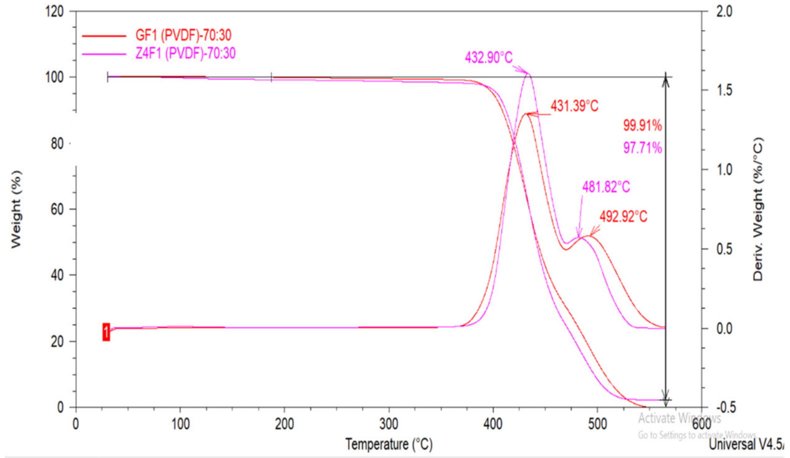

Temperature (°C)

Figure S5. Modulated thermogravimetric curves of the zeolite-free (GF1 red curve) and zeolite-doped (Z4F1 magenta curve) samples

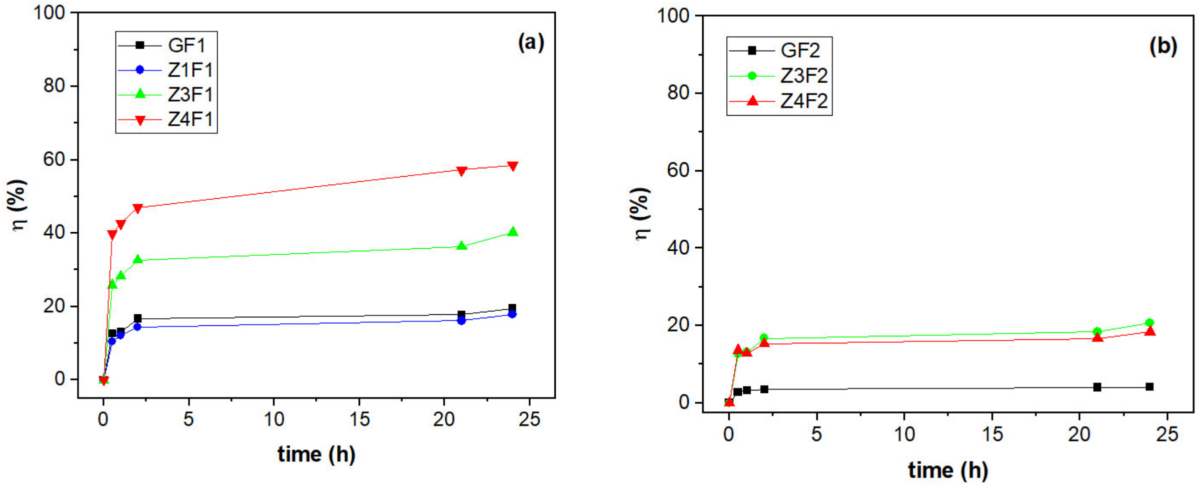

Figure S6. MB adsorption efficiency on the (a) F1 membranes and (b) F2 samples.

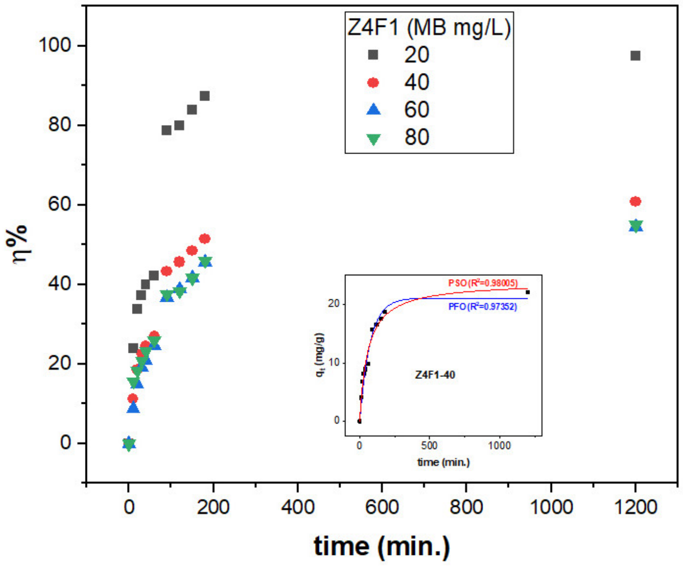

Figure S7. Adsorption efficiency of the Z4F1 sample at various MB concentrations (Inset stands for 40 mg/L MB adsorption kinetics on the Z4F1 sample)

Physio-sorption of MB on the Z4F1 sample was depicted at a low MB concentration of 20 mg/L, according to the PFO model fit of the q_t versus time data, while chemisorption of MB took place on the Z4F1 sample at richer MB concentrations (40, 60, and 80 mg/L) (Figure S7).

Table S1. Results of fit parameters (peak position/FWHM) and assignments [33,34] for the fibrous samples within the 700-950 cm^{-1} range, t and g, and are trans and gauche conformers.

GF2	GF1	Z4F1	Z4F2	Z3F1	Z3F2	Z1F1	Z0.5F1	PVDF powder	Assignments
774/42	771/54	772/34	772/43	775/27	772/38	772/21	770/19	770/24	CF2 and CCC scissoring
797/10.0	797/14.5	796/9	799/13.5	799/11.4	779/13.6	799/15.3	797/13.7	796/7.8	tg and tttg (t3g) sequences of α and γ phases
809/22	811/19	806/20	812/16	809/19	812/17	812/16	810/23	806/21	υ(CH2) t3g of γ phase
828/18	830/20	825/21	829/16	830/22	829/16	823/12	828/15	827/21	υ(CF2) in γ
841/10.4	841/8.6	840/10.3	842/9.9	842/9.4	841/9.2	840/15.5	839/9.7	841/9.4	υ(CF2) all-t of β phase
857/24	852/18		854/24	852/64	849/34	859/9	853/44	853/18	
875/10	870/7	874/8	876/12	878/8	876/5	878/16	875/12	875/8	υ(CC/CF2) in α,β and γ phases
882/11	882/17	880/13	884/15	884/14	882/18	882/24	883/15	883/21	υ(C-C/CF2) of β phase
889/27	896/22	889/23	898/20	894/24	892/29	888/24	899/14	903/15	
0.3489	0.3803	0.4389	0.5218	0.4728	0.4167	0.5504	0.3673	1.9114	I800/I840
0.9978	0.9802	0.9969	0.9977	0.9928	0.9976	0.9904	0.9962	0.9991	R2

Table S2. DSC fitted parameters (melting data) and crystallization degree (χ_m) of the zeolite-free PVDF-based samples, GF(1/2), and Z4F1 sample collected during a second heating cycle $\chi_m = \frac{H_m}{H_m^0} \times 100$ where H^0_m=104.7 J/g is the melting heat of full crystalline PVDF*

Sample	T_{on} (°C)	T_{pk} (°C)	T_{off} (°C)	H (J/g)	χ_m (%)
PVDF powder	158.56	167.99	171.80	49.816	47.57
GF2	160.40	170.58	174.73	45.149	43.12
GF1	160.77	169.83	174.37	56.178	53.65
Z4F1	160.30	169.36	173.90	57.338	54.76

*[61] Peng G., Wang L., Li F., Luo P. Structure and dielectric performance of poly(vinylidene fluoride)/organically modified montmorillonites nanocomposites. J. Elastom. Plast. 2015, 48 (3), 251–265. doi:10.1177/0095244314568473

The solvent effect on the GF2 sample is illustrated by its lower crystallinity degree.

Table S3 - Melting and Crystallisation Points (DSC data) collected during two successive heating-cooling cycles

**Sample**	**T_m-T_c Cycle 2 (°C)**	**T_m Cycle 2 (°C)**	**T_c Cycle 2 (°C)**	**T_m-T_c Cycle 1 (°C)**	**T_m Cycle 1 (°C)**	**Tc Cycle 1 (°C)**
PVDF 400k	36.38	167.6	131.22	35.28	166.5	131.22
GF1 (70/30)	34.19	170.1	135.91	33.27	168.9	135.63
GF2 (30/70)	35.35	169.7	134.35	33.15	167.5	134.35
Z4F1 (70/30)	33.91	169.4	135.49	33.64	168.7	135.06
